# Mechanical Properties, Radiation Resistance Performances, and Mechanism Insights of Nitrile Butadiene Rubber Irradiated with High-Dose Gamma Rays

**DOI:** 10.3390/polym15183723

**Published:** 2023-09-11

**Authors:** Rongrong Luo, Daoan Kang, Chao Huang, Tengfei Yan, Pengyuan Li, Hongxi Ren, Zhiyuan Zhang

**Affiliations:** 1 Southwestern Institute of Physics, Chengdu 610041, China; 2 Guizhou Aerospace Technology Control Co., Ltd., Guiyang 550025, China

**Keywords:** gamma irradiation, nitrile butadiene rubber (NBR), mechanical properties, radiation resistance properties

## Abstract

The radiation effect of materials is very important and directly related to the safety and reliability of nuclear reactors. Polymer materials, one of the indispensable materials in nuclear power equipment, must withstand the ordeal of high-energy ionizing rays. In this work, through screening different γ-ray dose irradiation conditions, we systematically and comprehensively study the changes in the structure and properties of nitrile butadiene rubber (NBR) before and after γ-ray static irradiation at a high dose rate, and master the rule and mechanism of the γ-ray static irradiation effect of these polymer materials. The mapping relationship between the macroscopic properties, microstructure, and irradiation dose of NBR is accurately characterized. With an increase in total irradiation dose, the C=C double bond reaction occurs, and the C≡N bond, C=C, and C=O participate in the hyper crosslinking reaction. The glass transition temperature (T_g_) increases with the cumulative irradiation amount. With the increased total irradiation amount, the degree of rubber cross-linking increases, causing an increased crystallinity and decomposition temperature. A growing amount of gamma irradiation causes the mechanical properties of the rubber to degrade simultaneously, increasing the shore hardness while decreasing the tensile strength and ultimate elongation at break. When the cumulative amount reaches 1 MGy, the ultimate elongation at break decreases significantly. A cumulative dose of radiation resistance of 4 MGy can be achieved by the samples. This work can provide theoretical and experimental support for the long-term stability of nitrile butadiene rubber and its derivatives in nuclear radiation fields and space radiation conditions.

## 1. Introduction

Humanity faces a challenging task in decommissioning nuclear facilities and disposing of nuclear waste. Highly radioactive environments pose a significant harmful risk to the human body. Therefore, operations in nuclear environments are difficult to perform manually, and require automation or remote operation technology [1] (such as special robots for nuclear environments). Rubber is commonly used in robot equipment and is widely used in seals, tires, damping structures, and cable sheathing and shielding [2,3]. As a polymer material, rubber is susceptible to radiation, and will produce cross-linking or degradation changes under high-energy radiation conditions, resulting in changes in the physical properties of the material [4,5]. Material radiation resistance is a critical consideration when designing robots or automation equipment for high-radiation environments. When the rubber is irradiated by gamma rays, molecular chain cross-linking and a fracture reaction will occur inside the rubber, and the comprehensive performance of the rubber is greatly reduced on the macro level [6,7,8]. The cumulative radiation amount of γ-rays can cause irreversible and permanent damage to the rubber. The cumulative damage caused by long-term irradiation to rubber may cause mechanical accidents, such as seal failure and leakage, posing a huge challenge to the safety of the entire system [9]. It is therefore important to consider the radiation resistance of rubber materials when designing robots and automation equipment for environments with high radiation levels.

Fusion robots are typically exposed to high dose rates, long periods of time, and a high cumulative total dose of radiation during their operation. The total radiation amount of the Blanket Remote Handling System (BRHS) of an International Thermonuclear Experimental Reactor (ITER) is 1 MGy after continuous operation for 180 days, and the cumulative γ-ray amount will reach 2.5 MGy if continuously operated for one year [10]. This cumulative radiation amount will directly affect the cross-linking and fracture of the molecular chains in the rubber, and then affect the comprehensive properties of the rubber in terms of chemical, mechanical, and electrical aspects. Actually, several rubber materials have failed to be applied after being irradiated by γ-rays with a cumulative total amount of 1 MGy [11].

Radiation shielding materials are widely used to mitigate the adverse effects of ionizing radiation sources, heavy metals, such as Fe, Pb, and W, have a good radiation protection efficiency for γ-rays, so as to decrease the weight of equipment, and polymeric composites are improved by adding metal or metal oxide particles as fillers [12]. At the same time, additional properties such as being lead-free, biocompatible, and environmentally friendly are needed for gamma-ray-shielding materials [13,14]. Atef et al. researched promising PVC/NBR/PbO shield blends for low gamma radiation doses [15]. Intom and his colleges successfully developed an excellent radiation shielding material based on NR/Bi_2_O_3_ composites [16]. Limarun and collaborators developed flexible, lightweight, and lead-free gamma-shielding materials from NR composites with additions of Bi_2_O_3_ and a blowing agent [17].

So far, researchers have reported the effects of various factors on the gamma radiation resistance of Nitrile Butadiene Rubber (NBR) and related rubbers. During high-energy radiation irradiation, the chemical structural of NBR changes due to the free radicals and ions being generated [18]. The chemical structure, additives, and irradiation dose of the rubber affect the degree of cross-linking, for instance, Vijayabaskar studied the influence of the electron-beam-initiated cross-linking of nitrile rubber with different acrylonitrile contents [19]. Research indicated that the polyfunctional monomers (PFMs) (such as antioxidant) on NBR are useful for obtaining the optimum mechanical properties at lower dose levels [20].

Carbon black and silica serve as reinforcing fillers used in the rubber industry for improving its properties [21,22,23]. Marković and coauthors investigated gamma irradiation (100, 200, and 400 KGy) on acrylonitrile butadiene/chlorosulphonated polyethylene rubber blend (NBR/CSM)-based nanocomposites containing carbon black and silica filler, and they showed that 30 phr of carbon black and 20 phr of silica provided the best gamma aging resistance to the NBR/CSM rubber nanocomposites [24]. Eyssa et al. explored the effect of different contents (5 to 20 phr) of silica fumed or sand, as well as the effect of gamma irradiation (25 to 150 KGy), on the properties of NBR nanocomposites, and the results showed that the presence of silica in the composites enhanced the electrical insulating properties of the NBR, with the properties improving up to 15 phr of silica content [25].

Moreover, NBR is a water-lubricated bearing material with excellent vibrational absorption and impact resistance properties and has been widely used in the production of ship stern bearings, e.g., Dong reported that nano-MoS_2_ reduced the friction coefficient of NBR significantly and reduced the friction noise [26]. Jafari studied the effects of electron-beam-irradiated poly (tetrafluoroethylene) (IR-PTFE) on the mechanical and tribological properties of carbon-black-filled NBR vulcanizates [27], but the literature does not contain any reports on gamma irradiation after adding lubricants of NBR.

NBR belongs to the cross-linking type of rubber when exposed to high-energy radiation; the degree of cross-linking seriously influences the properties of NBR [28], and, in most cases, cross-linking increases the strength and heat resistance while decreasing the elasticity, whereas the destruction of the polymer chains decreases the strength [29]. Shershneva explored the effect of gamma irradiation on industrial-grade NBR, and they found that the dose of gamma irradiation can impact the molar mass distribution of NBR, the molecular weight has a maximum of 10–20 KGy and decreases at higher doses, and the molecular weight rises constantly with an increasing irradiation dose; it was shown to provide a good understanding of radiation-induced cross-linking and the following processes in cross-linked polymers [30].

Although much progress has been made on NBR, most of the research regarding NBR polymer materials is not systematic, and the dose rate and cumulative dose of most studies are low, making it difficult for the materials to be selected for a high-radiation environment. In addition, it is rare to find a model correlation between the mechanical properties of the rubber and the radiation dose. In this work, the changes in the structure and properties of NBR before and after high-dose-rate γ-ray static irradiation were systematically and comprehensively explored by screening different high-γ-ray-dose irradiation conditions. The rule and mechanism governing the effect of the static gamma radiation on polymer materials were investigated. Furthermore, the internal relationship between the macroscopical properties, microstructure, and irradiation dose of NBR was disclosed. The results showed that NBR polymers can be used as radiation-resistant materials or radiation protection materials in high-radiation environments.

## 2. Experimental

### 2.1. Sample Preparation

NBR, with the commercial name NBR 5176S, was obtained from Beijing Institute of Aeronautical Materials (Beijing, China). It had an acrylonitrile content of 21 wt.%, a density of 1.2 g/cm^3^, Mooney viscosity (ML (1 + 4) at 100 °C) of 70, and thickness of 2 mm, and the recipe of this study also contained other additives.

**Figure 1:**
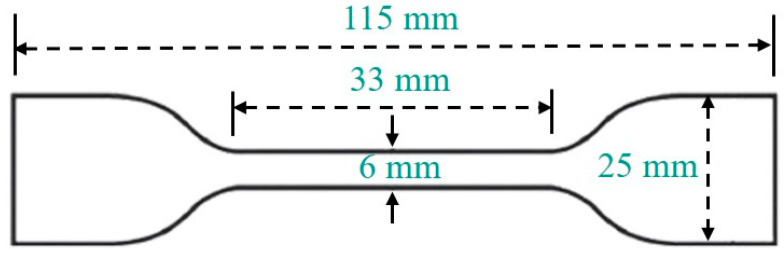
Dumbbell-shaped tensile sample.

A dumbbell shape was made, as shown in Figure 1. Then, the NBRs were irradiated by ^60^Co γ-rays; the irradiation was conducted with an FJ × 424 ^60^Co γ-rays irradiation device from the Sichuan Institute of Atomic Energy (Chengdu, China), with the irradiation amount of 50 KGy/h. The samples were taken out from the irradiation device when the cumulative amount reached 10 KGy, 100 KGy, 1 MGy, 2 MGy, 3 MGy, 4 MGy, and 5 MGy, respectively. These samples were named NBR76-0, NBR76-1, NBR76-2, NBR76-3, NBR76-4, NBR76-5, NBR76-6, and NBR76-7, respectively.

### 2.2. Characterization

Date containing the cross-linking degree of the NBR were measured using a MesoMR23/12-060H-I Magnetic Resonance Imaging (MRI) analyzer. The cross-link density (*v*) of the samples was determined using the theory of rubber elasticity, as follows:

(1) $$v=\frac{\rho \times 5N\sqrt{q}}{6CMru}$$

where ρ is the density of the NBR, N is the number of main-chain keys of repeating units, q is the anisotropy rate of the statistical segment, C is the number of main chains in the statistical segment, and Mru is the molar mass of the same structural units.

The tensile properties of the samples were tested according to GB/T 528-2009 [31]. The specimens were pulled to failure using a constant speed of 200 mm/min during the tensile test. On the other hand, the hardness measurements, conducted on unirradiated and irradiated samples to evaluate the mechanical properties of the rubber, were performed according to the GB/T 531.1-2008 [32] standard using the Shore A durometer (MEGA instrument, Chengdu, China).

Scanning electron microscopy (SEM) (FEI Company, Hillsboro, OR, USA) was used to characterize the cross-section morphology of the samples after stretching. A Empyrean X-ray diffractometer (PANalytical B.V., Almelo, The Netherland) was used to analyze the change in the diffraction peak of the fracture crystal, with a 2θ scanning range of 5–80°. The molecular structure was analyzed using Fourier transform infrared spectroscopy (FTIR) of Nicolet-5700 spectrometer (Thermo Electron, Waltham, MA, USA) in ATR mode, with an attenuated total reflection attachment and wavelength range of 500–4000 cm^−1^. The thermal properties and the glass transition temperature (T_g_) of the rubber materials were measured using a TGA/DSC3+ synchronous thermal analyzer (Mettler Toledo, Zurich, Switzeland). A thermogravimetric analysis (TGA) was performed with a Q2000 differential scanning calorimeter (TA instruments, Newcastle, DE, USA) in a nitrogen atmosphere.

## 3. Results and Discussion 

### 3.1. Structure and Morphology Characterizations

**Figure 2:**
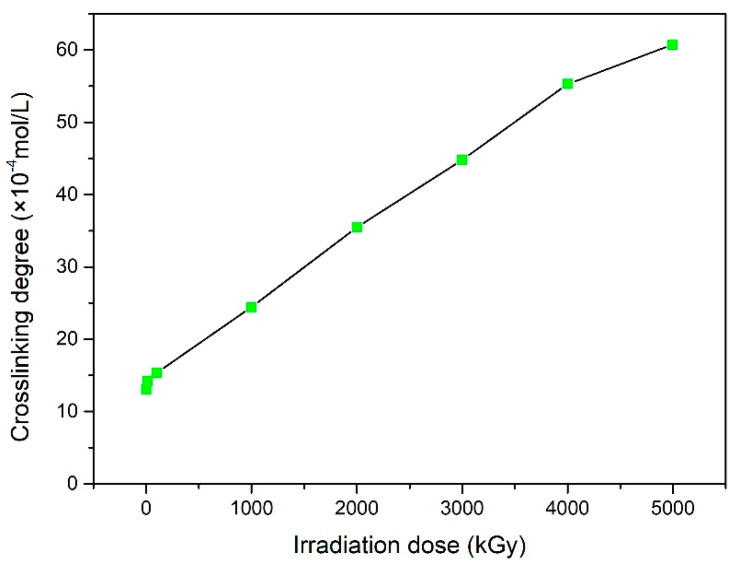
Change in the cross-linking degree of NBR with different γ-ray irradiation doses.

Figure 2 shows the change in the cross-linking degree between the samples before and after irradiation. When gamma-irradiated, NBR rubber undergoes a cross-linking reaction due to the increased dose of the total irradiation. Over the entire dose range studied, the cross-link density increased, in part due to the increased intermolecular radical–radical combinations and other rearrangements, which indicated the cross-linking behavior of the NBR polymers during irradiation [33].

**Figure 3:**
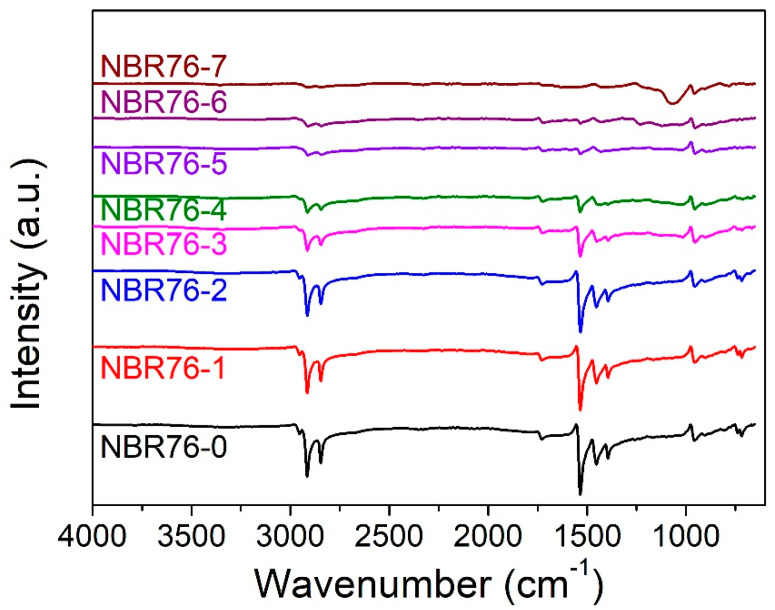
FTIR of NBR with different γ-ray irradiation doses.

FTIR was used to measure the infrared absorption spectrum of the rubber samples, determine the polymer molecular composition of the samples, and conduct a qualitative analysis of the functional group structure of the organic compounds, so as to judge the molecular structure changes in the sample before and after radiation. The FTIR results of NBR before and after γ-ray irradiation are shown in Figure 3. NBR is a copolymer of butadiene and acrylonitrile. The absorption peak near the wave number of 2237 cm^−1^ is a typical absorption peak for a cyano C≡N bond. The peaks at 990 cm^−1^, 967 cm^−1^, and 910 cm^−1^ were caused by the out-of-plane bending of the CH=CH_2_ of the NBR pyrolysis products, the peak at 967 cm^−1^ was caused by the out-of-plane bending of the trans CH=CH-, and the peak at 735 cm^−1^ corresponded to the absorption peak of butadiene’s cis−1,4 structure. It can be summarized that the bending vibration absorption peak of -CH_2_- was located at 1455 cm^−1^, the C=O vibration peak corresponded to 1534 cm^−1^, and the main structure of the molecular chains at 2913 cm^−1^ and 2839 cm^−1^ was a C-H bond, which were asymmetric and symmetric stretching vibration peaks of-CH_2_- distributed on the main chain. After γ-ray irradiation, typical butadiene and acrylonitrile peaks still existed, but other peaks gradually weakened or even disappeared. With the increased irradiation amount, the intensity of the infrared absorption peaks at 1434 cm^−1^, 2913 cm^−1^, and 2839 cm^−1^ gradually decreased and gradually disappeared after 1 MGy. This was because a C=C double-bond reaction occurred in the irradiation process of the NBR material and C≡N bond, C=C, and C=O participated in the high cross-linking reaction. The infrared absorption peaks associated with the C-H bonds and C=O bonds decreased with increasing the total irradiation dose; when the total irradiation dose reached 3 MGy, the cross-linking reaction intensified, resulting in their disappearance.

**Figure 4:**
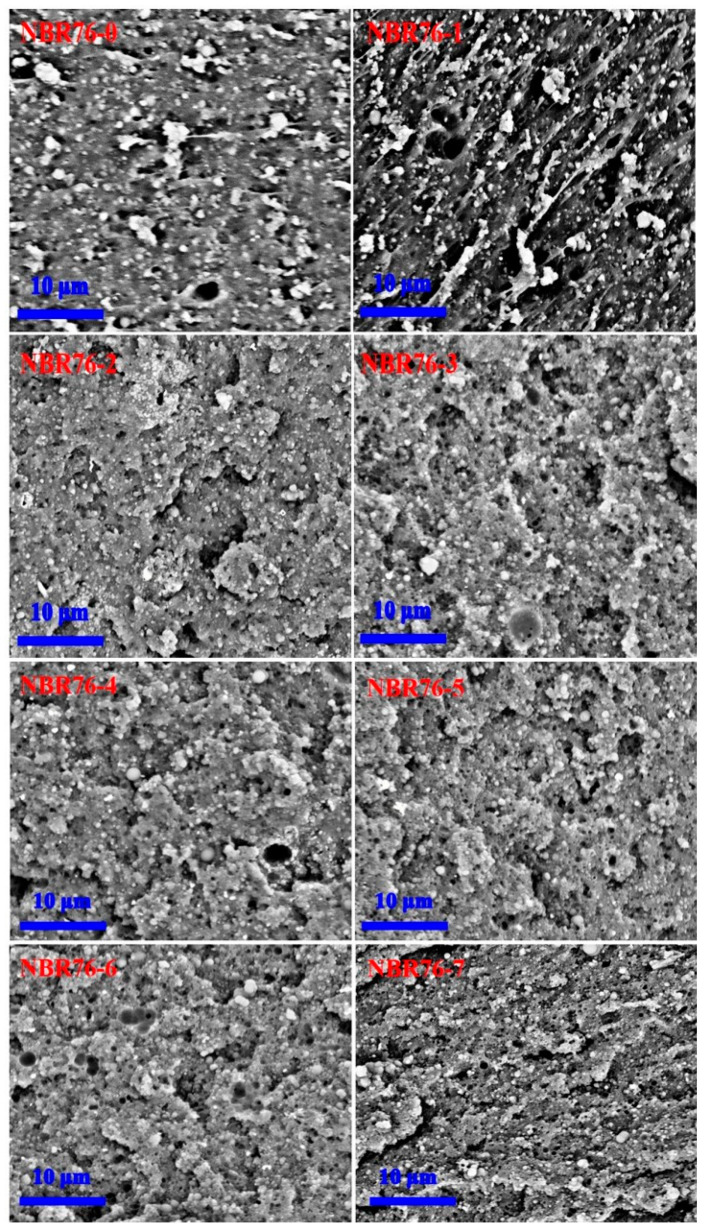
Cross-sectional SEM images of NBR with different γ-ray irradiation doses.

The cross-sectional morphology of the tensile NBR sample after different γ-ray irradiation amounts is shown in Figure 4. Compared with the unirradiated samples, gamma irradiation caused pores and cracks to appear on the NBR materials [18], and the fracture surfaces appeared deformed and morphologically rough, with numerous low ridges. The possible reason for this phenomenon was that γ-rays caused cross-linking reactions between the molecular chains, the relative molecular mass, and destroyed the microstructure of the polymer materials. After exposure to a total dose of 1 MGy, the irradiated surfaces exhibited lamellar fracture planes and small voids or pits. Due to the high gamma irradiation, the number of cavities or vacuoles and fracture morphology increased. It appeared that the cross-linking reaction dominated, though both crosslinking and degradation co−existed. Usually, they occur simultaneously, and one of them may be dominant, depending on the chemical structure and irradiation conditions such as the environment, dose rate, and temperature [34].

**Figure 5:**
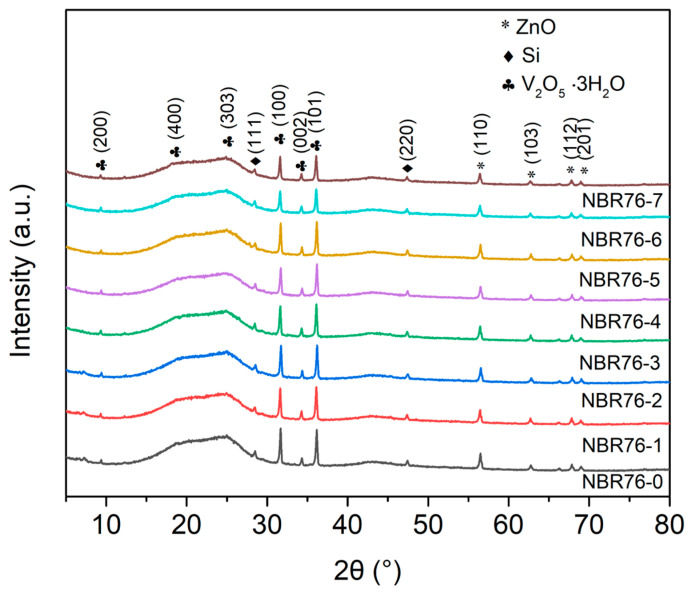
XRD patterns of NBR with different γ-ray irradiation doses.

Figure 5 shows the XRD patterns of the NBR with different γ-ray irradiation doses. The diffraction peaks were observed at 9.4°, 20.3°, 25.0°, 31.7°, and 36.2°, corresponding to the (200), (400), (303), (100), and (101) crystal planes of V_2_O·3H_2_O, respectively. The diffraction peaks positioned at 56.5°, 62.8°, 68.1°, and 70.3° corresponded to the (110), (103), (112), and (201) crystal planes of ZnO, respectively, while the peaks at 28.5°, 47.5°, and 34.3° corresponded to the (111), (220), and (002) crystal planes of Si, respectively. The diffraction signals of these crystals may have come from the additives of NBR.

It can be seen from the figure that the internal crystal structure of the rubber after irradiation did not change significantly, and the NBR samples showed obvious crystal peaks and amorphous peaks. The amorphous (Aa) and crystalline (Ac) areas of the polymer samples with different γ-ray irradiation doses were calculated in order to determine numerical values of the crystallinity (C). The crystallinity of the NBR samples with different irradiation doses was determined by the formula [19] to be 16.83%, 10.9%, 14.81%, 18.42%, 19.37%, 19.48%, 20.77%, and 20.19%.

C = Ac/(Аc + Aa) × 100%(2)

**Figure 6:**
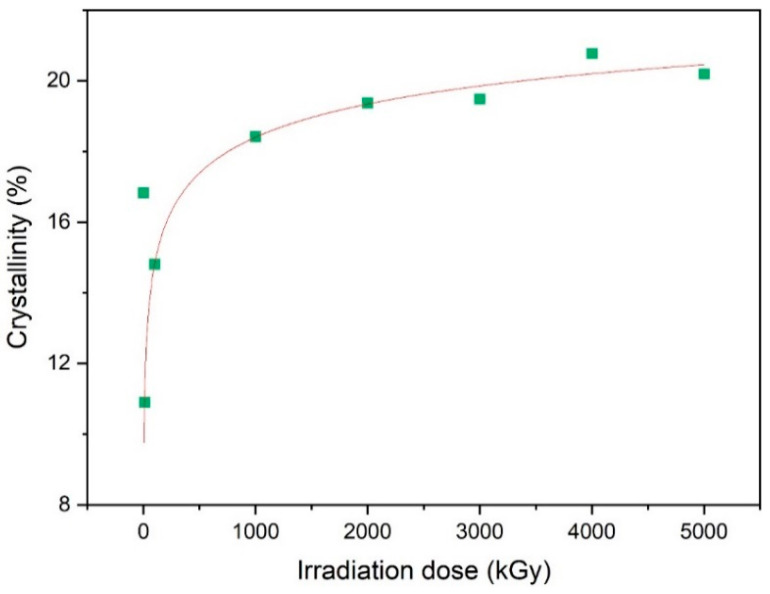
Dependence of crystallinity of NBR on different γ-ray radiation dose.

Figure 6 shows that, on the whole, the crystallinity of the NBR materials increased with an increase in the γ-ray irradiation dose. We suspect that the cross-linking caused by the irradiation occurred primarily in the amorphous region, and the edge crystal region adjacent to the amorphous region experienced grafting due to its looser molecular configuration, resulting in a less amorphous material and more crystallinity. We employed mathematical methods to fit a function model describing the relationship between the crystallinity of the NBR polymer and the radiation dose. The resulting model was found to be Lognormal CDF (Equation (3)), where y_0_ is 0.6685, A is 24.1851, and adjusted R-square is 0.9850.

(3) $$y=y_{0}+A\int_{0}^{x}\frac{1}{\sqrt{2\pi }wt}e^{-\frac{{\left(ln\left(t\right)-x_{c}\right)}^{2}}{2w^{2}}}$$

### 3.2. Thermal Properties

**Figure 7:**
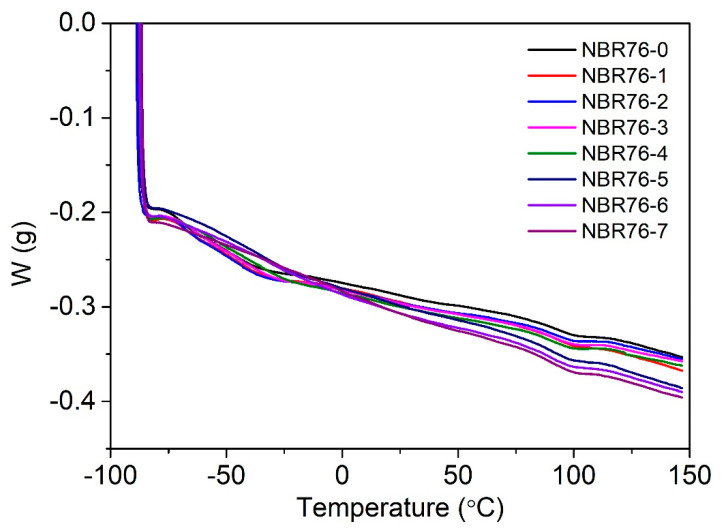
DSC curves of NBR with different γ-ray irradiation doses.

The DSC curves of the NBR with different γ-ray irradiation doses are shown in Figure 7, with the glass transition temperature (T_g_) shown in Table 1. The results show that the glass transition temperature (T_g_) of the non-irradiated NBR was −69.01 °C, and T_g_ increased with the increased irradiation amount after irradiation, which increased sharply after the doses increased to 1 MGy, showing that the cross-linking degree of the NBR76 rubber increased with the increased irradiation amount, resulting in an increase in T_g_. Moreover, it shows that the radiation cross-linking between the molecular chains in the rubber increased. The macroscopic properties show that the hardness increased and the ultimate elongation at break decreased.

**Figure 8:**
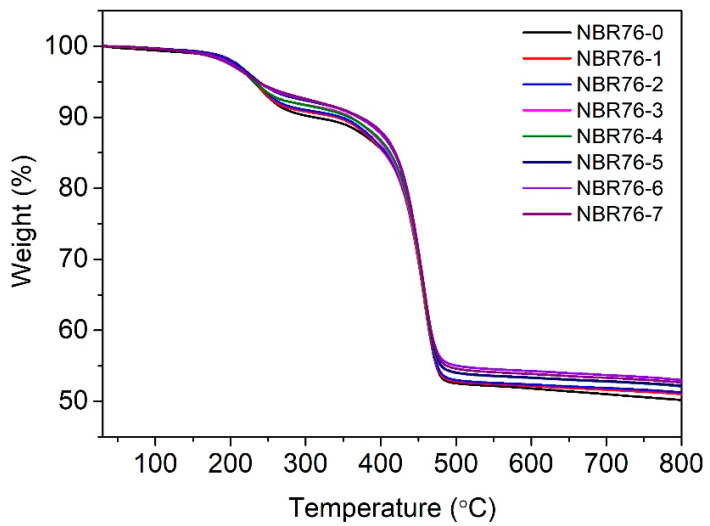
TGA curves of NBR with different γ-ray irradiation doses.

The TGA curves of the NBR with different γ-ray irradiation doses are shown in Figure 8. It was found that NBR76 began to decompose at 200 °C, the decomposition rate increased at 250 °C and reached its highest decomposition rate at 400 °C, and the decomposition ended at 475 °C, with a weight loss rate of over 50%. Rubber carbonization occurred as the total irradiation dose increased, and the carbonization rate increased. The residual carbon rate increased as the cross-linking density increased [35]. Meanwhile, the temperature increase from 10% degradation further indicated that γ irradiation promoted a cross-linking reaction.

The decomposition temperatures under different total irradiation amounts are shown in Table 2. Upon irradiation, the decomposition temperature of NBR decreased compared to that of the unirradiated samples, indicating a greater crystallinity and crosslinking as a result of the irradiation.

### 3.3. Mechanical Properties

**Figure 9:**
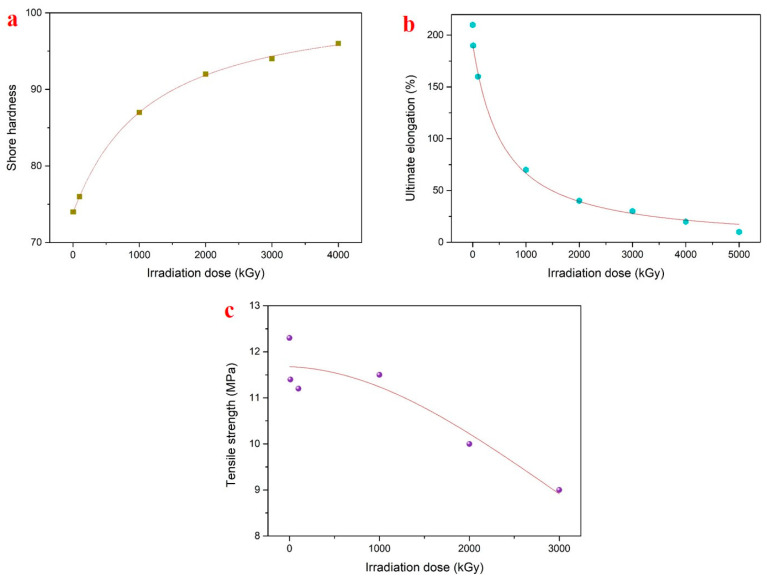
Mechanical properties of NBR with different γ-ray irradiation doses, (**a**) hardness, (**b**) ultimate elongation, and (**c**) tensile strength.

The changing curve of the shore hardness on the NBR surface with the cumulative γ-ray irradiation amounts is shown in Figure 9a. The results show that the hardness of the NBR sample increased with the increased irradiation amount. When the irradiation amount was 1 MGy, the hardness increased significantly, but when the cumulative irradiation amount reached 5 MGy, the sample became hard and brittle, failing to conduct hardness testing. During the irradiation process, the cumulative amount of γ-rays would cause irreversible permanent damage to the rubber.

**Figure 10:**
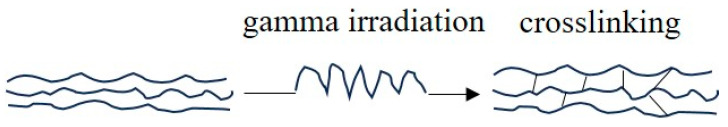
Schematic illustration of the cross-linking reaction of NBR.

In terms of the rubber irradiation reaction, it is worth noting that it is a complex process involving molecular chain degradation and cross-linking. With an increased cumulative irradiation amount, the degree of degradation and cross-linking reaction also changes. In the case of the NBR, cross-linking dominated when the irradiation dose was small, which increased the rubber’s hardness. When the irradiation amount was 100 KGy, the amplitude increased, and the molecular chains were excessively cross-linked and branched (Figure 10), resulting in the cyclization of the molecular chains and surface hardening and embrittlement. Nevertheless, after a cumulative dose of 4 MGy, the surface of the NBR became hard and brittle, and could not be measured.

The NBR materials of fusion robots will be affected by radiation irradiation when serving in high-radiation environments, which will lead to the deterioration of their properties. Therefore, the NBR samples were irradiated by γ-rays and the changes in the mechanical properties of the samples after irradiation were investigated. The curve of the ultimate elongation at the break and the tensile strength of the rubber with the irradiation amount is shown in Figure 9b,c; the ultimate elongation at break significantly decreased, rapidly decreasing at 1 MGy, which was essentially caused by the cleavage of molecular chains and the breaking of cross-linking bonds. The rubber’s tensile strength decreased gradually with an increasing irradiation dose; we speculate that, as the cross-linking density increased, the number of molecular chains bearing the load under tensile stress gradually increased, thus reducing the rubber’s tensile strength. After the cumulative dose reached 4 MGy, the NBR samples developed severe adhesion, causing the degradation reaction to increase and preventing the tensile test from being performed. When the rubber was subjected to a force, the changing cross-linking between the molecular chains created different interactions between molecules, affecting its macroscopic mechanical properties.

## 4. Conclusions

To summarize, when exposed to the irradiation of γ-rays, molecular chain cross-linking and fracture reactions will occur inside rubber. Meanwhile, a cumulative amount of radiation will influence the degree of cross-linking and fracture of NBR molecular chains, and further influence the comprehensive properties of rubber. The relationships between the crystallinity/mechanical properties of the NBR samples and irradiation dose were revealed. These findings contribute to a better understanding of the behavior of NBR under such high γ radiation dose conditions and could inform the screening of more robust polymer materials for radiation resistance applications.

## Figures and Tables

**Table 1:** Glass transition temperature (T_g_) of NBR with different γ-ray irradiation doses.

Sample	Irradiation Dose (KGy)	Tg (°C)
NBR76-0	0	−69.01
NBR76-1	10	−68.30
NBR76-2	100	−67.48
NBR76-3	1000	−63.71
NBR76-4	2000	−53.92
NBR76-5	3000	−36.31
NBR76-6	4000	−30.81
NBR76-7	5000	-

**Table 2:** Decomposition temperature of NBR with different γ-ray irradiation doses.

Sample	Irradiation Dose (KGy)	Decomposition Temperature (°C)
NBR76-0	0	432.85
NBR76-1	10	426.74
NBR76-2	100	427.07
NBR76-3	1000	427.94
NBR76-4	2000	426.98
NBR76-5	3000	430.80
NBR76-6	4000	430.08
NBR76-7	5000	430.17

## Data Availability

Not applicable.
